# Dual Vascular Insult of Fibrocartilaginous Embolism and Intercostal Artery Dissection Causing Anterior Spinal Artery Infarction in a 14-Year-Old

**DOI:** 10.7759/cureus.98871

**Published:** 2025-12-10

**Authors:** Sreeraj J, Parameswaran Krishnan, Anand M

**Affiliations:** 1 Neurology, Indo American Hospital, Vaikom, IND; 2 Neuroradiology, Indo American Hospital, Vaikom, IND

**Keywords:** anterior spinal artery infarct, fibrocartilaginous embolism, intercostal artery dissection, pediatric myelopathy, spinal cord ischemia

## Abstract

Acute spinal cord infarction (SCI) is a rare and devastating cause of myelopathy in adolescents. We report the case of a 14-year-old boy who developed acute paraplegia approximately 24 hours after a minor fall with axial loading. The clinical picture presented as the anterior spinal artery (ASA) syndrome, characterized by profound lower limb weakness and the pathognomonic finding of dissociated sensory loss (loss of pain and temperature with preserved proprioception). Magnetic resonance imaging (MRI) confirmed spinal cord ischemia with T2 hyperintensity restricted to the anterior cord. A subsequent aortic angiography revealed a concurrent right T10 intercostal artery dissection, establishing a definitive structural vascular injury. Given the classic clinical presentation (rapid onset, preceding localized pain) highly characteristic of nucleus pulposus fibrocartilaginous embolism (FCE), the final diagnosis was established as post-traumatic ASA territory infarction secondary to synergistic FCE and IC artery dissection. The patient was managed with neuroprotection, antiplatelet therapy directed at the dissection, and intensive rehabilitation. He achieved a remarkable recovery, ultimately regaining an independent and stable gait pattern. This case highlights the complexity of traumatic spinal vasculopathy and the importance of recognizing dual mechanisms of injury.

## Introduction

Acute myelopathy in adolescents presents a significant diagnostic challenge, most commonly arising from underlying inflammatory etiologies. However, vascular etiologies, while rare, must be promptly identified due to their distinct management and prognostic implications [1]. Spinal cord infarction (SCI) results from the occlusion of the anterior spinal artery (ASA), which involves disruption of the motor pathways and pain/temperature sensation [2].

​Two distinct and rare post-traumatic vascular mechanisms were involved in this case. The first mechanism, ​nucleus pulposus fibrocartilaginous embolism (FCE), results from the migration of disc material into the radicular arteries supplying the cord, a mechanism often precipitated by minor trauma or sudden axial loading [3]. This is particularly relevant in the bimodal adolescent age group, during which the disc’s vascular supply is regressing. The resulting embolic occlusion leads to acute ischemia. The second mechanism, intercostal (IC) artery dissection, involves a structural tear in the arterial wall that leads to hematoma formation and subsequent occlusion. If this occurs in a vital feeder vessel, such as the T10 IC artery (which often gives rise to the artery of Adamkiewicz, or AKA), it mechanically compromises the blood supply to the entire lower spinal cord. This report is significant because it documents the co-existence of a highly suspected FCE (based on clinical picture and exclusion of alternatives) and a confirmed, structural IC arterty dissection (based on angiography), presenting a unique dual-mechanism etiology for a single, catastrophic ASA infarct in a 14-year-old.

## Case presentation

The patient, a 14-year-old boy with no significant prior medical history, sustained a minor trauma involving a fall with presumed axial loading. After a 24-hour symptom-free interval, he experienced an abrupt onset of severe bilateral inner buttocks and posterior thigh pain. He presented to the Emergency Room on the next day after this pain had rapidly progressed over several hours to a maximal deficit of complete paraplegia and acute bowel and bladder incontinence. This rapid progression to maximal deficit is the hallmark of an acute occlusive event.

Neurological examination revealed an alert and oriented patient. Cranial nerve function and upper limb power were normal (5/5). Lower limb examination revealed profound hypotonia and paraplegia. Lower limb power was severely diminished (2/5). Reflexes were hyperreflexic, and plantar responses were bilaterally extensor. A positive Beevor’s sign localized the lesion to the T9-T11 segments. Sensory testing confirmed a definitive sensory level at D11 with dissociated sensory loss, with complete loss of pain and temperature sensation below D11 and preserved proprioception, vibration sense, and fine touch [2]. Given the constellation of symptoms at presentation, ASA infarction was suspected.

​Initial laboratory work, including inflammatory markers (ESR, CRP), was unremarkable helping to rule out an infectious or systemic autoimmune myelitis. Cerebrospinal fluid (CSF) analysis, performed to exclude infectious or inflammatory cell presence, was acellular with normal protein levels. Serological and CSF immunological testing for Myelin Oligodendrocyte Glycoprotein (MOG)-IgG and Neuromyelitis Optica (NMO)-IgG, performed to definitively exclude specific demyelinating disorders such as MOG antibody disease (MOGAD) and NMO Spectrum Disorder (NMOSD), was negative.

**Figure 1:**
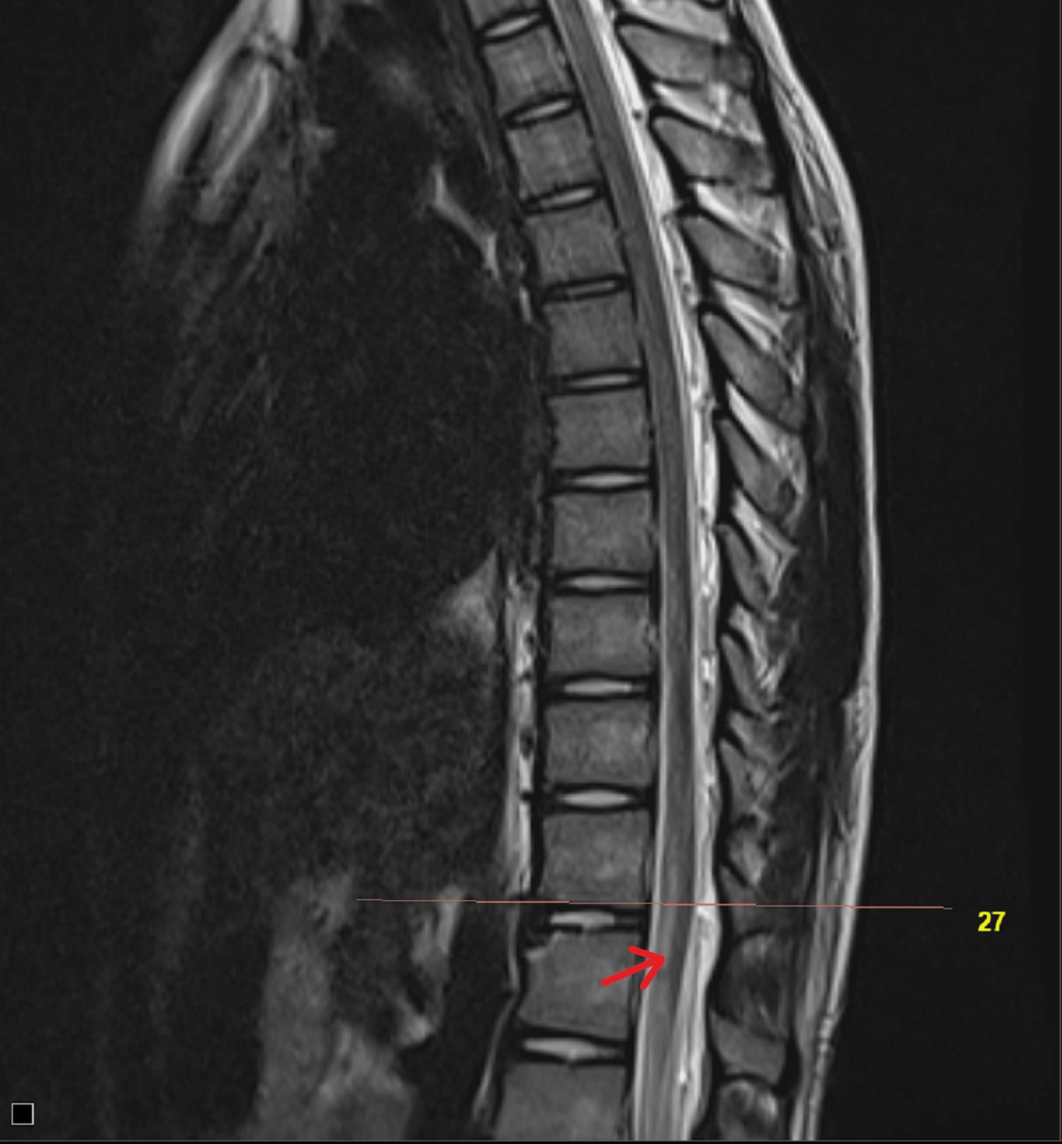
MRI T2 sagittal image of dorsal spine showing ASA syndrome MRI - T2 sagittal image of dorsal spine showing longitudinal ventral cord hyperintensity at D11 to L1 level (red arrow). MRI: Magnetic resonance imaging; ASA: Anterior spinal artery

**Figure 2:**
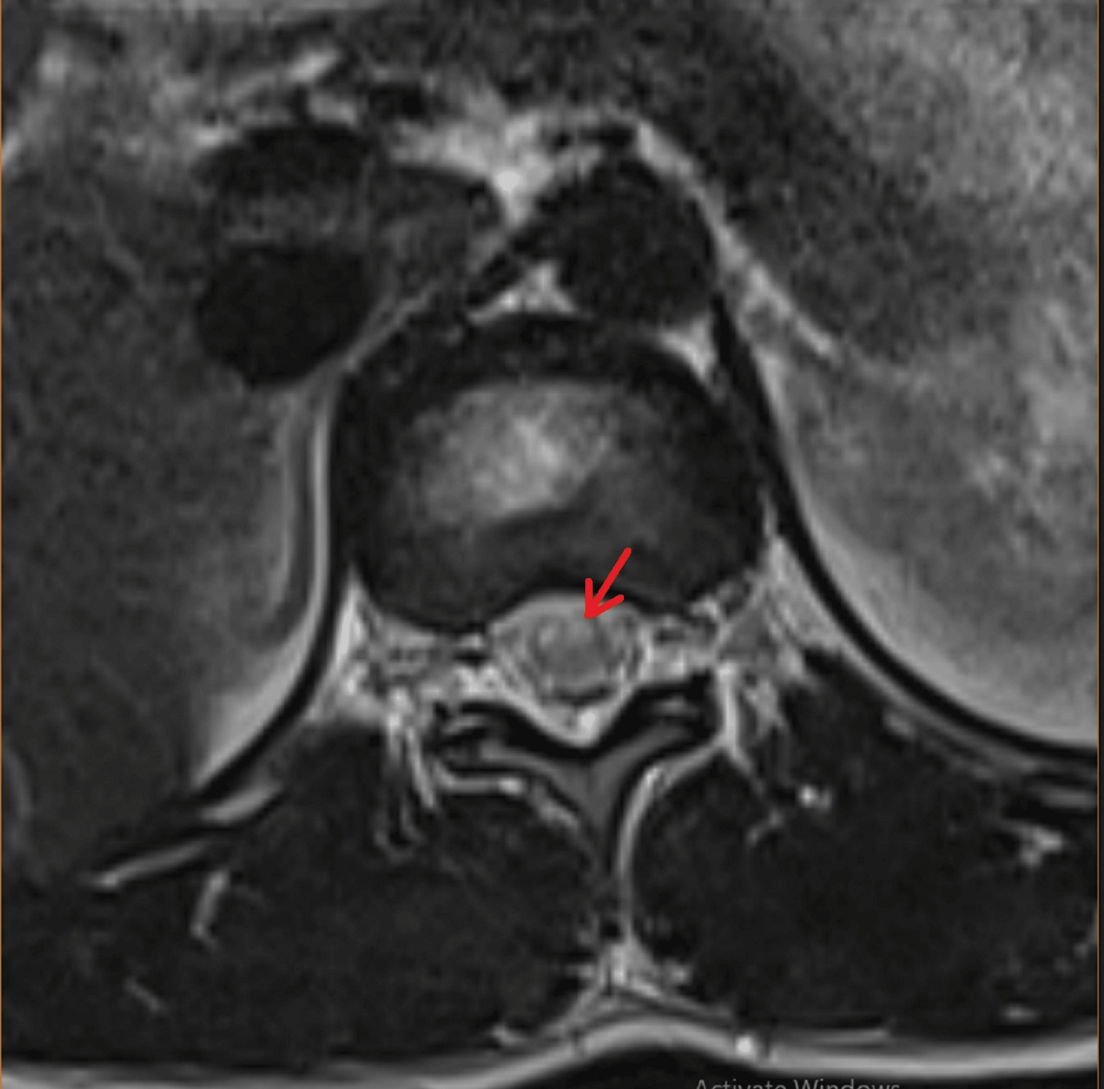
MRI T2 axial image of the same showing ASA syndrome MRI T2 axial image of the dorsal spine showed hyperintensity overlying bilateral anterior horn cell regions of spinal cord ("owl's eyes sign" or "snake eyes sign") corresponding to ASA territory Infarct (red arrow). MRI: Magnetic resonance imaging; ASA: Anterior spinal artery

A sagittal image of the magnetic resonance imaging (MRI) of the spine demonstrated longitudinal T2 hyperintensity restricted to the anterior two-thirds of the spinal cord parenchyma from the D11 to L1 level, consistent with an ASA territory infarct (Figure 1). An MRI T2-weighted axial image of the same region showed hyperintensity overlying the bilateral anterior horn cell regions of the spinal cord ("owl's eyes sign" or "snake eyes sign"), which is pathognomic for an ASA territory infarct (Figure 2).

**Figure 3:**
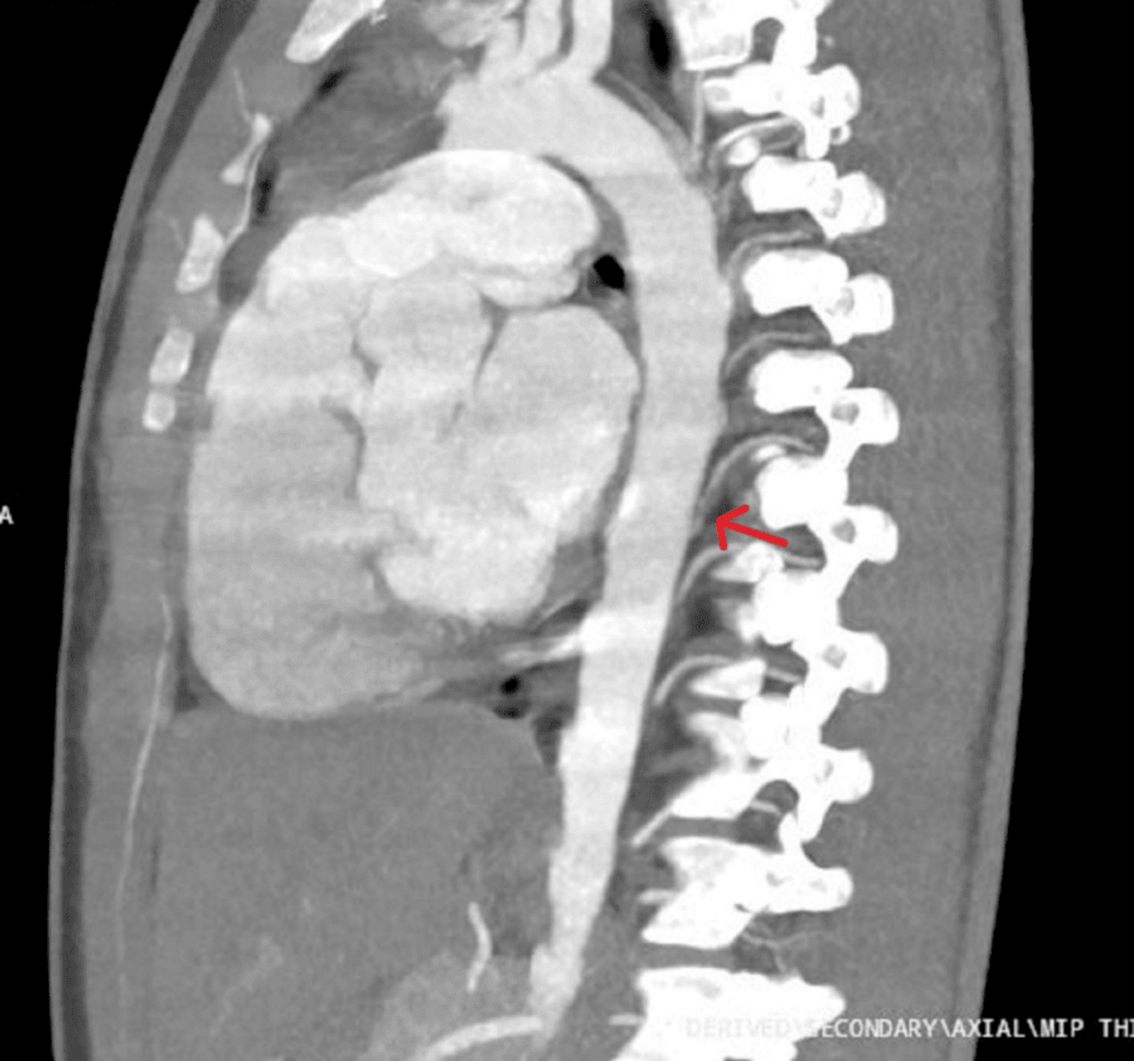
Oblique sagittal MIP showing D10 intercostal artery dissection Oblique sagittal MIP showing focal stenosis of D10 intercostal artery proximally due to intramural hematoma (dissection): red arrow MIP: Maximum Intensity Projection

**Figure 4:**
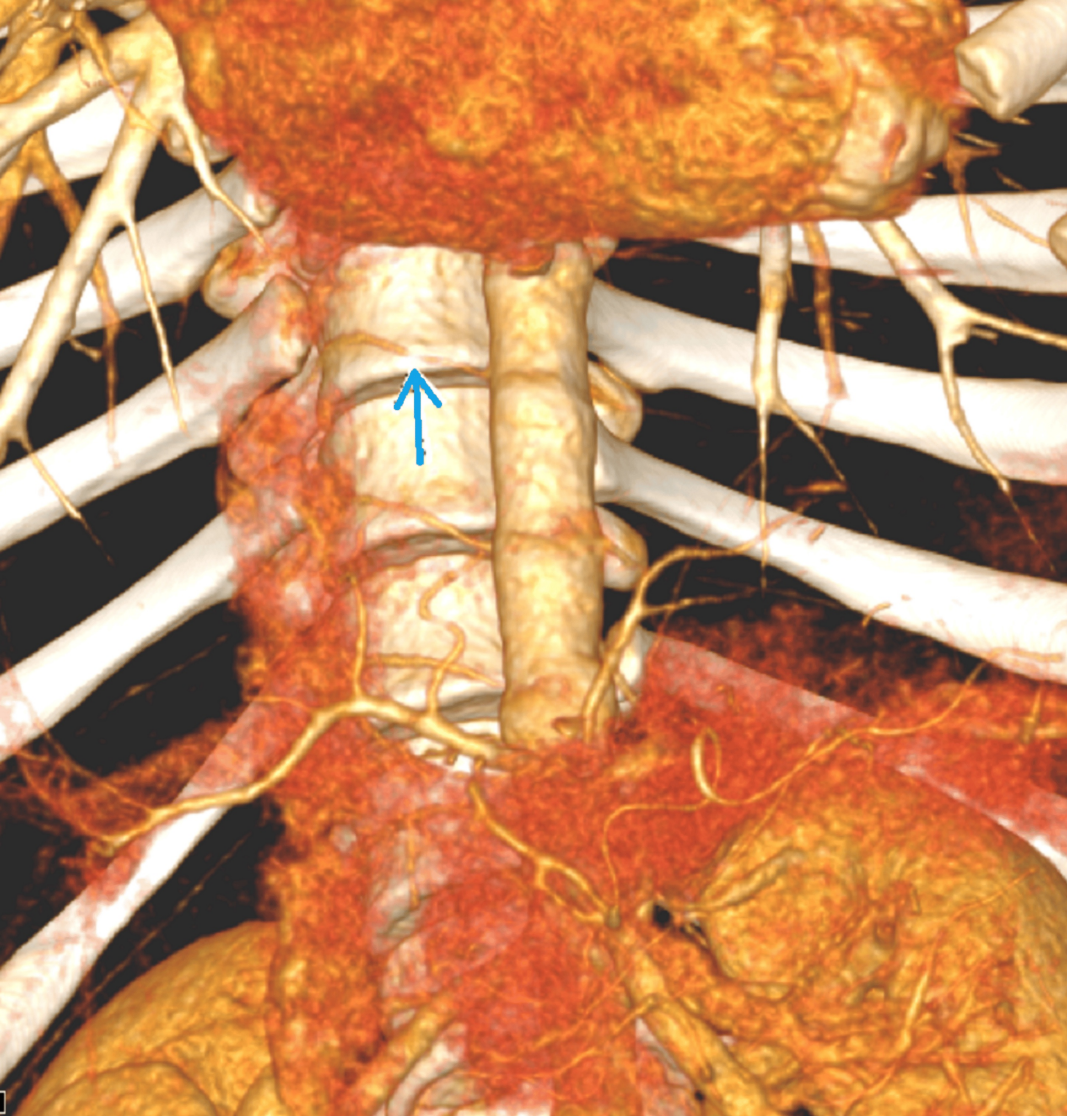
VRT aortogram showing D10 intercostal artery dissection VRT aortogram with arrows showing area of focal concentric narrowing secondary to dissection involving D10 intercostal artery (blue arrow) VRT: Volume rendering technique

An oblique sagittal maximum intensity projection (MIP) showed focal stenosis and narrowing of the right D10 artery proximal to the D10 IC artery hematoma (dissection) (Figure 3). A subsequent aortic angiogram using volume rendering technique (VRT) demonstrated focal concentric narrowing secondary to dissection involving D10 IC artery (Figure 4). This confirmed a right D10 intercostal artery dissection, representing a significant traumatic vasculopathy affecting a major ASA feeder vessel. 

The final diagnosis was post-traumatic ASA territory spinal cord infarct secondary to synergistic FCE and IC artery dissection. Acute management included high-dose intravenous corticosteroids and IVIg as neuroprotective agents, and to cover for an initial diagnosis of acute non-compressive myelopathy [4,5]. Due to the confirmed dissection, Aspirin 150 mg was immediately initiated and scheduled for a 6-month course to prevent thrombotic propagation [3,6,7]. The patient subsequently underwent intensive physical medicine and rehabilitation (PMR), with a focus on core strengthening and gait retraining. He demonstrated substantial functional recovery, achieving ambulation with an independent gait pattern upon discharge [8].

Differential diagnosis

​The primary objective of the initial diagnostic workup was to differentiate the vascular event from common inflammatory and compressive pathologies. Inflammatory and infectious causes were reliably excluded. Acute transverse myelitis and other demyelinating disorders were ruled out by the negative serology (MOG IgG, NMO IgG), acellular CSF, and the highly specific anterior cord-only signal abnormality on MRI, which is unlike typical inflammatory lesions. Infectious myelitis was similarly ruled out by the absence of systemic fever, normal inflammatory markers (ESR, CRP), and acellular CSF findings. Furthermore, spinal cord compression was excluded by the absence of any space-occupying lesion (such as a tumor, abscess, or large disc herniation) on the MRI. 

​The final differential diagnosis focused on the vascular mechanism. While pure IC artery dissection is a documented cause of spinal cord infarction, it was considered potentially insufficient as the sole etiology. This determination was based on the patient's strong clinical history, specifically the preceding, delayed-onset bilateral buttocks and thigh pain, which is highly suggestive of a micro-embolic event like FCE. This clinical alignment necessitated the final diagnosis of a synergistic dual mechanism.

## Discussion

​This case provides a unique demonstration of a severe ASA territory infarct resulting from a dual, synergistic vascular insult following minor trauma [9]. The simultaneous combination of a highly suspected FCE and a confirmed IC artery dissection elevates the clinical complexity and educational value of this case.

Rationale for proposing a dual mechanism

The clinical and anamnestic evidence strongly supported the inclusion of FCE in the diagnosis. The patient's presentation was characterized by a distinct 24-hour delay between the fall and the onset of symptoms, followed by a rapid progression. Crucially, the prodromal pain was localized to the buttocks and thighs (T10-L1 nerve root distribution). Importantly, this delayed, localized pain pattern is highly suggestive of the micro-embolic event of FCE, where disc material causes immediate irritation and initial ischemia of the small radicular arteries before lodging fully in the ASA [3,8].

Conversely, the right T10 IC dissection, confirmed on angiography, provided the definitive macrovascular trauma [10]. Since the T10 IC artery is a major supply route to the ASA via the artery of Adamkiewicz(AKA), the dissection served as a critical compounding factor, a macrovascular collapse, that prevented any collateral circulation from compensating for the microvascular occlusion initiated by the FCE [9,11]. Therefore, the devastating final ASA infarct was the result of the simultaneous failure of both microcirculation (embolus) and macrocirculation (dissection) [9,11].

​Clinical and therapeutic implications

The angiogram of the dissection was critical, as it directly influenced long-term management [12]. The confirmed arterial pathology necessitated the initiation of antiplatelet therapy (Aspirin) to prevent further thrombosis or propagation of the dissection, a treatment path not typically indicated for simple FCE [6,7,8]. The choice of aspirin was intentional and risk-appropriate. Given the acute spinal vascular event, minimizing the risk of hemorrhage was the primary clinical objective, providing necessary antithrombotic coverage to stabilize the dissection and prevent further clotting. Crucially, however, the evidence base for guiding antithrombotic therapy, specifically in acute spinal artery dissections, is extremely limited. Therefore, this management decision was an extrapolation, based on consensus guidelines for treating extra-cranial cervical artery dissections, where minimizing the risk of hemorrhage is paramount. Ultimately, this case emphasizes the vital importance of pursuing comprehensive vascular imaging (angiography) when spinal cord ischemia is suspected to guide specific medical interventions. Furthermore, the patient’s excellent recovery to independent gait underscores the high capacity for functional gain in younger patients following spinal stroke when prompt diagnosis is followed by aggressive, intensive PMR [8].

## Conclusions

This report describes an exceptionally rare dual-mechanism etiology for acute spinal cord infarction in a 14-year-old. Here, a suspected post-traumatic FCE occurred synergistically with a confirmed right T10 IC artery dissection, culminating in a catastrophic ASA infarct. This unique case reinforces that FCE should be considered when the clinical syndrome perfectly aligns, even when a confirmed, concurrent structural pathology (dissection) is present. The successful outcome highlights the critical role of specific medical therapy (antiplatelets) and aggressive physical rehabilitation in maximizing recovery potential following complex spinal vascular injury.
